# Tumour biomarkers: homeostasis as a novel prognostic indicator

**DOI:** 10.1098/rsob.160254

**Published:** 2016-12-07

**Authors:** Michela Falco, Giuseppe Palma, Domenica Rea, Davide De Biase, Stefania Scala, Massimiliano D'Aiuto, Gaetano Facchini, Sisto Perdonà, Antonio Barbieri, Claudio Arra

**Affiliations:** 1 Struttura Semplice Dipartimentale Sperimentazione Animale, Istituto Nazionale Tumori ‘Fondazione G. Pascale’, IRCCS, Via Mariano Semmola, 80131 Naples, Italy; 2 Molecular lmmunology and Immuneregulation, Istituto Nazionale per lo Studio e la Cura dei Tumori, IRCCS Naples ‘Fondazione G. Pascale’, Naples, italy, Istituto Nazionale Tumori ‘Fondazione G. Pascale’, IRCCS, Via Mariano Semmola, 80131 Naples, Italy; 3 Division of Breast Surgery, Department of Breast Disease, National Cancer Institute, IRCCS, ‘Fondazione Pascale’, Naples, Italy; 4 Division of Medical Oncology, Department of Uro-Gynaecological Oncology, , Istituto Nazionale per lo Studio e la Cura dei Tumori ‘Fondazione G. Pascale’, IRCCS, 80131 Naples, Italy; 5 Department of Urology, Istituto Nazionale per lo Studio e la Cura dei Tumori ‘Fondazione G. Pascale’, IRCCS, 80131 Naples, Italy; 6 Department of Veterinary Medicine and Animal Production, University of Naples ‘Federico II’, Via Delpino 1, 80137 Naples, Italy

**Keywords:** homeostasis, cancer, biomarkers, metabolomics

## Abstract

The term ‘personalized medicine’ refers to a medical procedure that consists in the grouping of patients based on their predicted individual response to therapy or risk of disease. In oncologic patients, a ‘tailored’ therapeutic approach may potentially improve their survival and well-being by not only reducing the tumour, but also enhancing therapeutic response and minimizing the adverse effects. Diagnostic tests are often used to select appropriate and optimal therapies that rely both on patient genome and other molecular/cellular analysis. Several studies have shown that lifestyle and environmental factors can influence the epigenome and that epigenetic events may be involved in carcinogenesis. Thus, in addition to traditional biomarkers, epigenetic factors are raising considerable interest, because they could potentially be used as an excellent tool for cancer diagnosis and prognosis. In this review, we summarize the role of conventional cancer genetic biomarkers and their association with epigenomics. Furthermore, we will focus on the so-called ‘homeostatic biomarkers’ that result from the physiological response to cancer, emphasizing the concept that an altered ‘new’ homeostasis influence not only tumour environment, but also the whole organism.

## 1. Introduction

The last decade has seen significant advances in the development of biomarkers in oncology; they play a critical role in understanding molecular and cellular mechanisms which drive tumour initiation, maintenance and progression. A cancer biomarker refers to a substance or process that is indicative for the presence of tumour in the body and therefore it may be a molecule secreted by the tumour or a specific body response to it [1]. Genetic, epigenetic, proteomic and imaging biomarkers can be used for cancer diagnosis, prognosis and epidemiology, and some of them can be assayed in organic fluids like blood or serum [2]. While numerous challenges exist in translating biomarker research into the clinic, a number of genes and protein-based biomarkers have already been used for patient diagnosis and care, including BRCA1/BRCA2 (breast-related cancer antigens), BRAF-V600E (melanoma/colorectal cancer), CA-125 (cancer antigen in ovarian cancer), CA19.9 (cancer antigen in pancreatic cancer), CEA (carcinoembryonic antigen in colorectal cancer), EGFR (epidermal growth factor receptor in non-small cell lung carcinoma), HER-2 (human epidermal receptor in breast cancer), PSA (prostate-specific antigen in prostate cancer) and many others [3–6].

Several biomarkers may be used not only to screen for primary tumour or patients prognosis, but also for monitoring status of disease, recurrence and response to therapy [7].

Currently, cancer biomarker research is rapidly growing to elucidate the molecular pathways for inter-individual differences in drug response. Recent technologies and their application, in the field of cancer therapy, have enabled identification of genetic variations that may predict patient response to chemotherapy and targeted therapies [8,9]. These genetic variations, together with epigenetic alteration (like DNA methylation and chromatin/histone modifications), can contribute to develop some new biomarkers [10,11].

## 2. Biomarkers in cancer

Tumour biomarkers are substances present in or produced by a tumour or by the microenvironment in response to tumorigenesis or progression processes. They can be virtually used in early cancer diagnosis, anti-cancer therapy development, monitoring of treatment responses and detecting individual risk for cancer development; for example, a woman that, during a screening, shows to be carrier of a germline mutation, such as BRCA1, has an increased risk of developing breast/ovarian cancer [12,13]. They can be used also to obtain other important information about the various aspects of the relationship between cancer and patient. Cancer biomarkers allow predicting the response to therapy, by evaluating the probable benefits of a particular treatment selected on the basis of the clinical information given by the biomarkers. In this way, the choice of the appropriate treatment leads to the development of increasingly personalized anti-cancer therapies [14] (table 1). There are several distinct types of tumour biomarker based on different tumour aspects: genetics, epigenetics, proteomics, metabolomics and imaging technology.

**Table 1.** Predictive and prognostic oncological biomarkers of solid tumours.

malignancy	predictive biomarker	gene abnormality	drug therapy	biological role of biomarker
colorectal	EGFR	over-expression	imatinib	signalling protein downstream of primary target
	K-ras G13D	gene mutation	cetuximab	
	B-raf V600E DPD	gene mutation	panitumumab	
breast	no mutated gene	none	tamoxifen	primary target
	ER/PR	gene deletion/absence of expression	aromatase inhibitor	drug metabolism
	BRCA1/2	mutation or deletion	olaparib	predictive and prognostic biomarkers
	HER2/neu (Erb-B2)	gene amplification	trastuzumab	
NSCLC	EGFR	over-expression	gefitinib	DNA repair
	ERCC	gene mutation	erlotinib	downstream of primary target
	K-ras	gene mutation	platinum biological	
prostate	PSA	over-expression	enzalutamide	blocking testosterone
	PCA3	gene mutation		

## 3. Colorectal cancer

Colorectal carcinoma (CRC) is the most common cancer of the gastrointestinal tract and the second most frequently diagnosed malignancy in adults [15]. Treatments used for CRC may include some combination of surgery, radiation therapy, chemotherapy and targeted therapy. Most recently, biologic agents such as cetuximab/panitumumab (monoclonal antibodies directed against the epidermal growth factor receptor, EGFR) and bevacizumab (a humanized monoclonal antibody that targets vascular endothelial growth factor) have been proven to have therapeutic benefits in CRC alone or in association with standard chemotherapy [16].

Randomized controlled trials (RCTs) have shown that colon screening is associated with a reduction in CRC mortality. In fact, some screening detects cancer at an early stage, when treatment is less arduous and more often results in cure, while other screening has the ability to detect adenomas as well as cancer [17]. CRC is a disease in which pathogenesis is influenced by genetic and epigenetic events that occur with tumour initiation and progression. Any biomarkers that have been identified can be used to predict clinical outcome beyond staging, and to inform treatment selection [18].

The improvements in early detection, thanks to the screening and the use of prognostic biomarkers, have led to a decline in the incidence rate of colon cancer for the past 2 years [17]. In clinical routine biomarkers [19] such as EGFR gene expression, K-ras G13D gene mutation, BRAF-V600E gene mutation are considered for therapy (table 2).

**Table 2.** Genetic biomarkers in colorectal cancer patients.

biomarkers	therapy
EGRF	anti-EGFR monoclonal antibody
KRAS	cetuximab and panitumumab
BRAF	monoclonal antibody

## 4. Clinical biomarkers in colorectal cancer

### 4.1. Human epidermal growth factor receptor 2

Human epidermal growth factor receptor 2 (HER2) is a member of the EGFR family, having tyrosine kinase activity. Approximately 70% of human colorectal cancers express EGFR protein. Receptor dimerization results in the auto-phosphorylation of tyrosine residues within the cytoplasmic domains of the two receptors, and in the initiation of a variety of signalling pathways leading to cell proliferation and tumorigenesis. Therapies directed against HER2 have revolutionized the treatment of HER2 overexpressing CRC and gastric cancers, and they have improved their clinical outcome. Anti-EGFR monoclonal antibodies (mAb), such as cetuximab and panitumumab, competitively inhibit EGFR by preventing its binding to endogenous ligands [20,21].

### 4.2. K-ras (G13D gene mutation)

K-ras, a member of RAS proto-oncogenes family, is the most frequently mutated gene in all human cancer and particularly it is an important oncogene in CRC. The K-ras protein is a downstream effector of EGFR that signals, through BRAF, the mitogen-activated protein kinase (MAPK) pathway activation and promotes cell growth and survival [22]. Mutations in K-ras codons 12 or 13 occur in approximately 40% of colorectal cancers and lead to constitutive signalling by impairing the ability of GTPase activating proteins to hydrolyse K-ras-bound GTP; these mutations cause resistance to cetuximab and panitumumab [23]. Recently, published RCTs have established the use of K-ras mutational analysis as a predictive marker for anti-EGFR mAb resistance in patients with metastatic colorectal cancer [24,25].

### 4.3. BRAF (V600E gene mutation)

Currently, BRAF mutations are found in 35–45% of colorectal cancers and they are considered to be a prognostic biomarker for poor prognosis in patients receiving first-line colon cancer therapies [26]. The biological evidence for BRAF-V600E mutations employment as an additional biomarker of anti-EGFR mAb resistance is strong: (i) BRAF is the immediate downstream effector of K-ras in the Ras/Raf/MAPK signalling pathway and (ii) BRAF-V600E activating mutations are 100% mutually exclusive of K-ras mutations in colorectal cancer, implying that the activation of either protein is sufficient for colon tumorigenesis. Previous studies support the use of BRAF-V600E as a negative predictor of response to anti-EGFR mAb therapy, leading to the evolving use of BRAF mutation testing in K-ras/wt patients [27]. This is considered to be an emerging biomarker of negative response to K-ras [28].

## 5. Epigenetic biomarkers in colorectal cancer

CRC occurs in most cases as a result of both mutations and epigenetic modifications accumulated in several genes, particularly DNA mismatch repair genes, which cause the progression of disease from early adenoma to carcinoma and eventually to metastatic disease. In CRC, the hyper- or hypo-methylation events have been observed at each histological step from the polyps to adenocarcinoma [29].

Hyper-methylation events on CpG islands affect virtually all signalling pathways, including those of TP53, TGFβ/SMAD, WNT, NOTCH and tyrosine kinase receptors as well as those involved in cell cycle and transcription regulation, DNA stability, apoptosis, cell-to-cell adhesion, angiogenesis, invasion and metastasis [30,31]. Conversely, hypo-methylation, characterized by the gradual and complete depletion of methylated cytosine bases (5-methyl-cytosine) in cancer cells, is observed even in early stages of CRC until its development and progression [32].

Many studies have investigated the potential role of expression genes for prognostic use, and unsurprisingly most of them are similar to those with a high potential for diagnostic use; in example, promoter CpG methylation of HLTF and CDKN2A is used with prognostic and diagnostic functions in tumours [33,34]. More recent studies have revealed additional epigenetic biomarkers linked to CRC staging and progression.

Methylation levels of genomic repeats, such as long interspersed nuclear element (LINE-1), have been recognized as independent factors for increased cancer-related mortality. LINE-1 hypo-methylation constitutes a potentially important feature of early onset CRC, and suggests a distinct molecular subtype [35]. Early onset of CRC represents a clinically distinct form of CRC that is often associated with a poor prognosis. LINE-1 enhanced activation through hypo-methylation is associated with increased genomic instability and enhanced cancer ability to penetrate surrounding tissues and metastasize [36,37].

## 6. Breast cancer

Breast cancer (BC), the most common cancer among women, is a heterogeneous and complex disease, whose precise progression mechanisms are less understood [38]. The molecular subgroups, also known as intrinsic subtypes of BC, have been defined by gene expression profiles, and they have distinct clinical features, metastatic behaviour, prognosis and treatment [39]. Despite the subtype identification, inter- and intratumour heterogeneity remain the principal causes of the marked differences observed in patients' response to therapy and their prognosis [40] (table 3).

**Table 3.** Clinical biomarkers for breast cancer.

molecular subtype	biomakers	treatment
hormone receptor	Ki67 index	tamoxifen
	hormone receptor expression	
	loss of ER positivity	
HER2+	loss of HER2	monoclonal antibody
	gain of ER positivity	
triple negative	gene mutations	CMF or CEF adjuvant chemotherapy

Neo-adjuvant therapy (NAT) has become one of the standard treatments of patients with locally advanced BC; it allows reduction of the tumour mass before surgery. NAT can be used to turn a tumour from untreatable to treatable by decreasing the volume. The tumour burden reduction after treatment with NAT influences disease-free survival (DFS), or rather the length of time after treatment during which no disease is found [41].

According to American Society of Clinical Oncology (ASCO) recommendations, tumour biomarkers like oestrogen receptor (ER), progesterone receptor (PR) and HER2 expression should be evaluated in primary invasive BC for diagnosis, disease recurrence and especially as a guide for therapy, while increasing levels of CA27.29 or CA15-3 may indicate treatment failure [42].

## 7. Clinical biomarkers in breast cancer

### 7.1. Oestrogen receptor and progesterone receptor gene expression

The status of a BC is routinely identified by immunohistochemistry through identification of both predictive and prognostic biomarkers [43]. ER-positive status has the best predictive value for DFS [44], whereas PR-positive status indicates the presence of a functionally intact oestrogen response pathway, but it has primarily a prognostic and not predictive value compared with pharmacological treatment with tamoxifen. Moreover, high expressions of eR and PR are predictive for benefit from hormonal therapy in adjuvant treatment in patients with metastatic disease (Stage VII disease). Current clinical guidelines suggest that hormonal therapy is recommended for all patients with ER-positive disease regardless of their level of ER [45,46], even if not all ER-positive metastatic BCs respond to it. Recently, some reports have shown a genomic index for sensitivity to hormonal therapy based on genes associated with ESR1 (DNA copy of the ER) [47].

### 7.2. HER2 (Erb-B2)

HER2 is a gene overexpressed or amplified in 15–30% of invasive BCs, and it has both prognostic and predictive implications with a reduced survival [44]. HER2-positive tumours show an over-expression of HER2 protein, which has a predictive value compared to therapeutic treatment in patients of newly diagnosed BC. Moreover, over-expression of HER2 protein also shows a favourable response in patients treated with Trastuzumab, a monoclonal antibody that targets and blocks HER2 receptor, improving progression free survival and disease control. Oppositely, HER2-negative tumours do not respond to Trastuzumab [48,49]. In addition, there is new evidence that BC patients with HER2-positive tumours often benefit from Topoisomerase II (encoded by TOP2A gene) inhibitor-based chemotherapy such as doxorubicin or epirubicin [45].

## 8. Epigenetic modifications as biomarkers in breast cancer

Genetic and epigenetic alterations can control cancer induction and progression. Epigenetics refers to alterations in gene expression due to modifications in histone acetylation (HDAC) and DNA methylation of the promoter regions of genes. In BC biopsy specimens, HDAC-1 is associated with ER and PR expression; its gene expression levels gain during the earlier stage of neoplasia, representing a good marker of improved DFS [50]. HDAC-6 messenger RNA (mRNA) is more frequently expressed in ER- and PR-positive BC patients with small lesions (less than 2 cm) and low aggressiveness grade. However, different analyses failed to confirm that HDAC-6 expression is an independent prognostic factor for survival [50].

In BC, CpG island methylations of gene promoter regions play a major role in regulation of gene expression involved in a large spectrum of biological processes. Aberrant DNA hypo- or hyper-methylation should be useful as prognostic or diagnostic markers.

DNA methylation in RASSF1A, DCR2APC and PTEN genes is observed in snap-frozen primary breast tumour associated with different stages of BC progression [51]. Therefore, DNA hyper-methylation of PITX2 (paired-like homeodomain transcriptor factor-2) was recently considered as a marker linked to tamoxifen response [52].

A recent study [53] assessed methylation levels of CpG islands promoter of tumour suppressor genes, RARb2, MINT17 and MINT13 during key steps of BC development. They have showed that DNA hyper-methylation of selected biomarkers occurs early in BC development, and may present a predictor of malignant potential [53].

Different epigenetic profiles have also been identified in hormone receptor-positive and -negative tumours [54–56]. The methylation of HIN-1 and RASSF1A strongly correlate with ER and/or PR expression, whereas RIL and CDH13 methylation closely link to negative ER and/or PR. Subsequent studies have shown that the differences of methylation profiles between hormone receptor-positive and -negative breast tumours can also influence tumour response to hormonal therapy like tamoxifen [56,57].

## 9. Lung cancer

Lung cancer (LC) is the most common reason of cancer deaths and [58,59] about 85% of LCs are non-small cell lung cancers (NSCLCs), traditionally divided into three major cell types: adenocarcinoma (≈50%), squamous cell carcinoma (≈35%) and large cell carcinoma (≈15%). The overall 5-year survival rate for LC has risen only 4% (from 12 to 16%) over the past 4 decades, and late diagnosis is a major obstacle in improving LC prognosis [60]. The most common symptoms are coughing (including coughing up blood), weight loss, shortness of breath and chest pains [61].

The presence of biomarkers in the plasma of patients with LC has aroused great clinical interest, since, with a simple blood test, a valid biomarker could be used for screening, diagnosis, prognosis, progression assessment and monitoring of therapeutic response [62]. A number of diagnostic biomarkers for LC have been suggested [63], including carcino-embryonic antigen, neuron-specific enolase, Cytokeratin 19 (CYFRA-21.1), alpha-feto protein, serum carbohydrate antigen-125 (CA-125), carbohydrate antigen-19.9 (CA-19.9) and ferritin. These biomarkers have varied sensitivities for different subtypes of LC [64,65].

The major advance in the treatment of NSCLC developed from the recognition that specific genetic alterations define subsets of NSCLC; these subsets are characterized by genetic and molecular alterations in the EGFR [66]. However, the lack of a uniform approach to extraction and quantification has made the standardization of any particular biomarker difficult [67].

## 10. Clinical biomarkers in lung cancer

### 10.1. The epidermal growth factor receptor

EGFR is a 170-kDa plasma membrane glycoprotein consisting of a large extracellular region, a single transmembrane domain and an intracellular domain with tyrosine kinase activity and a C-terminal tail. The EGFR family consists of four closely related receptors: HER-1/ErbB1, HER-2/neu/ErbB2, HER-3/ErbB3 and HER-4/ErbB4 with significant homology in their kinase domains, but differences in the coding regions for the extracellular domain and the C-terminal tails [68]. The molecular analysis of mutations in EGRF gene, its corresponding downstream signalling cascade and the related mutations have led to the development of novel therapies [69]. Data from this biomarker, when combined with analysis of histological material, are becoming very important in LC diagnosis as well as in patient stratification for therapy. EGFR is a widely used therapeutic target to treat patients with NSCLCs. There are mutations that are specific to NSCLCs that activate EGFR. They are deletions in exon 19 and exon 21 point mutation, L585R. These mutations result in ligand-independent activation of EGFR signalling [68]. Two irreversible anti-EGFR tyrosine kinase inhibitors are currently approved for the treatment of advanced NSCLC (gefitinib and erlotinib). Recent phase III randomized trials with these EGFR inhibitors, when compared with chemotherapy, have produced significantly longer DFS, higher response rates, less toxicity and a better quality of life. The ‘combination affinity’ of increased gefitinib and erlotinib with the mutated form of EGFR is expected to represent an approximately threefold improvement over that likely from chemotherapy alone in unselected NSCLC patients [68,70–72].

### 10.2. K-ras (gene expression)

K-ras is the most commonly detected mutation in NSCLC. It is more common in tumours with adenocarcinoma histology than in squamous-type NSCLC. K-ras mutation was previously considered a negative predictive biomarker for efficacy of EGFR targeted inhibitors, but, to date, there is no targeted therapy with established efficacy in NSCLC for this genetic mutation. Therefore it does not offer, at present, any clinical value either as a prognostic indicator or as a therapeutic guide. Currently, targeted therapies against activating K-ras mutation are undergoing active testing as a therapeutic strategy in LC [73,74].

## 11. Epigenetic modifications as biomarkers in lung cancer

LC involves an accumulation of genetic and epigenetic events in the respiratory epithelium [75]. Somatic genetic aberrations, such as mutations and copy-number alterations, play a well-known role in oncogenesis, but epigenetic alterations are more frequent than somatic mutations in LC [76]. LC initiation and progression are due to the interaction among genetic, epigenetic and environmental factors. The DNA hipo- or hyper-methylation is the most widely form of epigenetic alteration in LC; the presence of hypermethylated gene increases with neoplastic progression from hyperplasia to adenocarcinoma. Many studies have identified a plethora of hypermethylated promoter genes such as RASSF1 [77], CDKN2A [78,79], CYGB [80], RARβ [81], APC [77,82], FHIT [83]. RASSF1A is deleted or methylated in 30–40% of NSCLC and 70–100% of SCLC; FHIT is deleted or methylated in 40–70% of NSCLC and 50–80% of SCLC [84]. Methylation of RASSF1A gene combined with K-ras mutation is reported to be a good marker of prognosis in detection of malignancy in false-negative or ambiguous cytology outcomes [85,86].

## 12. Prostate cancer

In many countries, prostate cancer (PCa) is the second most frequently diagnosed cancer in males and the second cause of malignancy-related death. The rate of PCa increases significantly after 40 years and about two-thirds of all prostate cancers occur in men 65 years and older [74,87].

PCa may have various clinical courses with different features including slow-growing tumour with no clinical consequences, or rapid development which leads to aggressively metastatic and lethal outcome [88]. The main therapy for patients with metastatic or progressive disease targets androgen production and its mediator, the androgen receptor (AR). These therapies, known as hormonal or androgen ablation treatments, refer to the administration of anti-androgens that block the functional action of AR [89]. Differently from other tumours, PCa biomarkers are usually serum or urine markers, because there are not specific molecular mutations that may be used for prognostic or diagnostic aims.

The introduction of PSA has revolutionized PCa screening, and it has ushered in the PSA era; its employment as diagnostic biomarker has allowed an earlier PCa detection, showing an increased incidence. However, its use as a screening tool remains controversial due to unresolved questions about survival benefits, cost effectiveness, and some clinical factors such as the optimal screening age or the PSA levels at which to recommend biopsy [90].

## 13. Clinical biomarkers in prostate cancer

### 13.1. Prostate-specific antigen

PSA, also known as gamma-semino protein or kallikrein-3, is a kallikrein-like serine protease; a glycoprotein enzyme encoded by an androgen-responsive gene (19q 13.3–13.4). PSA is secreted by the epithelial cells of the prostate gland [91] and it is produced for the ejaculate, where its main role, thanks to the proteolytic function, is to liquefy semen in the seminal coagulum, allowing sperm to swim freely [92,93]. PSA is generally present in small quantities in the serum of men with a healthy prostate, while its levels are often elevated in the presence of PCa or other prostate disorders; for these reasons PSA is the only biomarker that is used for diagnosis and prognosis of prostate tumour [94]. The large use of the PSA test has increased disease detection at earlier stages [95], allowing a decrease in the number of patients in metastatic state [96]. PSA biomarker has been also used as a staging and prognostic tool as its high levels are found in more progressive stages or in more unfavourable result [97]. In spite of this significant role, PSA is organ-specific but not cancer-specific, and therefore it is not a unique indicator of prostate tumour. In fact, serum PSA levels also increase in benign prostatic hyperplasia, in size of prostate secondary to a non-cancerous proliferation of prostate gland cells [93], in the prostatitis (inflammation of prostate), in following interventions like biopsy [98], in older age, in ejaculation and in the use of specific drugs such as male hormones. So only 30% of patients with high PSA have PCa diagnosed after biopsy. Besides, there are several factors that may cause decrease in PSA levels, including 5-α reductase inhibitors, herbal mixtures, obesity, aspirin, statins and thiazide diuretics [99]. One of the main limitations of the PSA test is hence represented by the false positives. Recent data showed that a substantial number of men had PCa with PSA values in the normal range and many of these patients had a high-grade malignant disease [92]. Over the last years, all these observations have impaired the association between PSA and PCa [100,101], and in order to increase PSA diagnostic specificity and prognostic ability, other parameters (such as percentage of free PSA or PCA3) are now increasingly using.

### 13.2. Percentage free prostate-specific antigen

Serum PSA is present in different molecular forms that can be divided into two classes: free PSA (not bound) and complex PSA (bound to protease inhibitors such as α1-antichymotrypsin, α1-antitrypsin, α2 macroglobulin) [97,102]. Free PSA represents 5–45% of total PSA. Its percentage is calculated by free PSA/total PSA × 100, and it has been considered as an appendix to total PSA testing, in men with a serum total PSA value of 4–10 ng ml^−1^ [96]. Many studies suggest free PSA as a late-stage predictor of PCa [103] and in particular the percentage of free PSA seems to be inversely associated with risk of finding PCa in biopsy [104]; the researchers show that percentage of free PSA is significantly low in aggressive disease conditions like Gleason score ≥ 7, metastases or positive surgical margins [105]. Gleason score is one of the most important predictors of disease outcome. It is a prognostic grading system based only on histological pattern of differentiation and organization of carcinoma cells and its values can change from 2 to 10 [106]. It is found that by using a percentage of free PSA cut-off value of 25%, it is possible to detect PCa with 95% sensitivity and to prevent 20% of unnecessary biopsies [105]. Therefore, percentage of free PSA could be a better predictor of post-operative pathological outcome when compared with Gleason grade [107], even if this opinion has not been confirmed [108,109].

### 13.3. PCa antigen 3

Urine-based PCa assays have been regarded as a promising tool for the acquisition of highly specific prostatic markers. PCa antigen 3 (PCA3) mRNA expression levels within post-digital-rectal-examination urine have been evaluated as predictors for the PCa detection on subsequent biopsy, whereby higher expression levels of PCA3 have been associated with PCa discovery [110]. A urinary PCA3 assay (Progensa, Hologic Inc., Bedford, MA, USA) is currently approved by the Food and Drug Administration in the setting of prior negative biopsy, where different studies have examined the predictive value of using PCA3 thresholds to select men for repeat biopsy [111].

## 14. Epigenetic modifications as biomarkers in cancer prostate

Epigenetic modifications are heritable and reversible biochemical changes of chromatin structure [112–117]. Unlike mutations that involve an alteration in the DNA sequence, epigenetic modifications regulate gene expression via chromatin remodelling [5]. Among the most well-studied epigenetic modifications are DNA methylation and histone modifications. Epigenetic alterations are frequent in PCa, and they can contribute to the tumour initiation and progression [118]. Although the mechanisms by which these alterations arise are not completely understood, their frequency is commonly higher in premalignant disease stages, giving them an attractive role for diagnosis, prognosis and treatment [6,119–121]. DNA methylation patterns may be the earliest changes in PCa and in effect, many studies have identified a promoter CpG island hyper-methylation of genes, such as GSTP1, APC, RASSF1α, PTGS2 and RARβ2; this evidence proposes that multigenes promoter methylation testing could be necessary. A multicentre study has validated the use of three gene panel (GSTP1, APC and RARβ2) as a diagnostic maker for PCa [122–125], and moreover several approaches have shown the potential use of PTGS CpG island hyper-methylation as an important tool for recurrence risk prediction [126].

### 14.1. TMPRSS2-ERG

A chromosomal rearrangement in PCa has been identified and associated with earlier precancerous lesions; it is the TMPRSS2-ERG, fusion gene between transmembrane protease serine 2 (TMPRSS2) and v-ets avian erythroblastosis virus E26 oncogene homologue (ERG). Measurement of the TMPRSS2-ERG in urine, using quantitative nucleic acid amplification, has been evaluated as a marker, with high specificity for PCa, for disease in the pre-diagnosis setting. The combination of PCA3 levels with TMPRSS2-ERG measurement may offer improved discrimination of disease on biopsy [127,128].

### 14.2. Glutathione-S-transferase P1 (GSTP1)

This gene encodes an enzyme required for DNA detoxification and for its protection from oxidants and electrophilic metabolites, is a potential epigenetic biomarker due to its high specificity (more than 80%) compared with PSA serum. Several studies have focused on the use of GSTP1 as potential diagnostic or/and prognostic biomarker. GSTP1 hyper-methylation levels can be correlated to different disease stages or recurrence risk after treatment and its presence in serum, plasma and urine could be used to screen men when the value of other biomarkers is borderline. However, despite being highly specific, it appears to have a low sensitivity (18–40%) [129–131].

## 15. Homeostatic biomarkers and role in cancer prediction

The human body constantly interacts with the external environment that exposes it to several natural and artificial agents; they can produce irreversible damage or reversible imbalance of homeostatic processes causing various diseases, including cancer. Homeostasis alterations can influence the function of epigenetic regulation, tissue architecture and immune system play [132–134].

Homeostasis is a complex process due to the continuous monitoring of several physiological parameters and functions (such as the blood pressure, temperature, acid–base balance and water–salt balance) that are regulated to maintain human body stability; so that the cells can continue to live and work regularly in a suitable environment to their needs [58].

Changes in the homeostatic balance can influence fluid composition; therefore, an environmental alteration (volume and physical–chemical composition) activates the homeostatic mechanism to correct such imbalance and to re-establish all parameters (volume of water, the concentration of ions, hormones, osmotic pressure, oxygen tension and pH) within ‘physiological’ range of values. This mechanism allows a ‘new homeostasis’ inside the tumour due to the cancer cells' ability to adapt to the environment, establishing new balances, different from previously altered ones. The homeostatic switch can be evaluated monitoring different indexes: metabolic, neuroendocrine, immune and physiological parameters [135].

These parameters can be correlate with tumour progression and they can be considered as prognostic disease markers. The metabolic alterations are the first changes that occur in oncological patients; the typical parameters of this new condition are lactate, enzymatic activities, oxidative stress biomarkers, NOS/NO, cholesterol and many others [136,137]. Acidosis, for example, is common in cancer, for which homeostatic markers of this condition may be represented by the metabolic enzymes such as LDH or pH parameters like pH extracellular values (Phe), representative of the known Warburg effect (i.e. the phenomenon in which tumour cells rely mainly on glycolysis for energy production even in the presence of sufficient oxygen, which is the most outstanding characteristic of energy metabolism in cancer cells [138,139]). Cancer cells employ this altered metabolism to sustain a high proliferation rate [140]. The lactate dehydrogenase-A that catalyses the inter-conversion of pyruvate and lactate is the main enzyme responsible for the Warburg effect, thus it is upregulated in human cancers and associated with aggressive tumour outcomes [141]. Therefore, in cancer, many studies have targeted the glycolytic pathway, and in particular LDH enzyme, with the aim to develop or to screen new innovative anti-cancer strategies [142,143].

Changes in tumour pHe values can be assessed by different molecular imaging techniques such as ^64^Cu PET-based imaging, hyperpolarized MRI or acid CEST MRI. Importantly, several studies have shown a correlation between anti-cancer metabolism targeted therapies and reduced growth rate or apoptotic responses, so pHe may be also used, during treatment, as a biomarker for determining drug efficacy and much sooner than detecting a reduced tumour volume with morphological imaging [144].

The TCGA (The Cancer Genome Atlas) project using next-generation sequencing has profiled the mutational status and expression levels of all the genes involved in diverse cancers, including those that have a role in cholesterol metabolism, showing the role of the cholesterol pathway in cancer development and supporting a correlation between these genes and the disease prognosis [145].

Neuroendocrine system participate in disease development; the main biomarkers can be catecholamine, ACTC, glucocorticoids, neuropeptide Y, prolactin and serotonin [146]. Homeostatic responses can involve localized body regions or the whole body. The nervous system is one of the main homeostatic regulation systems, whose alterations could affect its specific control functions; some of these alterations could be represented by stress or depression conditions. Usually, these conditions are more frequent in oncologic patients. Stress or depression conditions influence tumour growth and metastasis development. For these reasons, indirect homeostatic biomarkers, such as epinephrine, norepinephrine and cortisol, can be evaluated. In effect, different studies have demonstrate, *in vitro* and *in vivo*, that higher stress hormones can influence proliferation rate, migration, tumour growth and metastasis; these data have also been confirmed by the use of beta blocker agents, suggesting the role of stress markers in the prognosis in various cancer [147–149].

The evaluation of inflammatory/immunity indexes and physiological parameters (cardiac frequency, VO_2_ max, body temperature and EEG) is important to determinate the complete oncological patient status both in diagnosis and prognosis. For this reason, during follow-up, it is important to check inflammatory profile (PCR, VES, neutrophilia, cytokines, urinary pH), immune outline, oxidative stress markers (endogenous and exogenous antioxidants) and other homeostatic parameters beyond specific molecular disease markers. The prognostic value of these markers is fundamental to evaluate every phase of the pathology progression and treatment response, with the aim to adopt personalized therapies and improve lifestyle, and to improve the patient healing.

## 16. Discussion and conclusion

Translational research on tumour biomarkers has successfully promoted new strategies for therapeutic treatment of cancer, instilling new hopes for cancer patients [150]. Biomarkers can influence the diagnosis and, consequently, the treatment of almost every patient with cancer. Thus, particular emphasis needs to be directed to the clinical approach, which will provide researchers with a critical point of view to improve solutions for patients. The development of new drugs requires high levels of attention and every compound needs to be tested in carefully designed and randomized clinical trials prior to governmental approval. Unfortunately, similar requirements are not mandatory for biomarkers, although they too can significantly influence patient outcomes. Therefore, it is important for clinical, translational and laboratory-based researchers to be acutely aware about the importance of the appropriate biomarker, in order to introduce them in clinical practice. In addition, the introduction of biomarkers that have not been sufficiently evaluated should be avoided because they could not only be ineffective, but even potentially detrimental to patient care. The initial conditions of cancer begin as an imbalance between the instability of the body system and the homeostatic mechanisms. In normal condition, the balance between proliferation and programmed cell death, usually by apoptosis, is strictly maintained by a fine regulation of both processes that ensure the integrity of organs and tissues. Mutations in DNA produce dysregulation and impairment of these regulatory processes, and subsequently lead to cancer. However, genomic and epigenomic alterations do not contemplate the countless interactions of homeostatic processes that occur in every living organism. In our opinion, cancer should not be considered as an indistinct entity in an organism, but as a strongly connected entity with the body itself. Most importantly, we should improve the diagnostic and therapeutic approach, also considering those markers of homeostasis that are indices of the operation of the body system *in toto*. We therefore propose a medicine no longer genomic-centric but human-centric.
